# An Analysis of Arterial Pulse Wave Time Features and Pulse Wave Velocity Calculations Based on Radial Electrical Bioimpedance Waveforms in Patients Scheduled for Coronary Catheterization

**DOI:** 10.3390/jcdd12070237

**Published:** 2025-06-20

**Authors:** Kristina Lotamõis, Tiina Uuetoa, Andrei Krivošei, Paul Annus, Margus Metshein, Marek Rist, Sulev Margus, Mart Min, Gert Tamberg

**Affiliations:** 1 Angiography Department, East Tallinn Central Hospital, 10138 Tallinn, Estonia; 2 Cardiac Intensive Care Unit, Tartu University Clinic, 50406 Tartu, Estonia; 3 Confido Healthcare Center, 11313 Tallinn, Estonia; 4 Thomas Johann Seebeck Department of Electronics, Tallinn University of Technology, 19086 Tallinn, Estoniamargus.metshein@taltech.ee (M.M.); mart.min@taltech.ee (M.M.); 5 Angiography Department, Tartu University Clinic, 50406 Tartu, Estonia; 6 Division of Mathematics, Department of Cybernetics, Tallinn University of Technology, 19086 Tallinn, Estonia; gert.tamberg@taltech.ee

**Keywords:** electrical bioimpedance, blood pressure, cardiovascular risk, wearables, pulse arrival time, pulse wave velocity, complementary pulse wave velocity

## Abstract

The monitoring of peripheral electrical bioimpedance (EBI) variations is a promising method that has the potential to replace invasive or burdensome techniques for cardiovascular measurements. Segmental or continuous recording of peripheral pulse waves can serve as a basis for calculating prognostic markers like pulse wave velocity (PWV) or include parameters such as pulse transit time (PTT) or pulse arrival time (PAT) for noninvasive blood pressure (BP) estimation, as well as potentially novel cardiovascular risk indicators. However, several technical, analytical, and interpretative aspects need to be resolved before the EBI method can be adopted in clinical practice. Our goal was to investigate and improve the application of EBI, executing its comparison with other cardiovascular assessment methods in patients hospitalized for coronary catheterization procedures. Methods: We analyzed data from 44 non-acute patients aged 45–74 years who were hospitalized for coronary catheterization at East Tallinn Central Hospital between 2020 and 2021. The radial EBI and electrocardiogram (ECG) were measured simultaneously with central and contralateral pressure curves. The Savitzky–Golay filter was used for signal smoothing. The Hankel matrix decomposer was applied for the extraction of cardiac waveforms from multi-component signals. After extracting the cardiac component, a period detection algorithm was applied to EBI and blood pressure curves. Results: Seven points of interest were detected on the pressure and EBI curves, and four with good representativeness were selected for further analysis. The Spearman correlation coefficient was low for all but the central and distal pressure curve systolic upstroke time points. A high positive correlation was found between PWV measured both invasively and with EBI. The median value of complimentary pulse wave velocity (CPWV), a parameter proposed in the paper, was significantly lower in patients with normal coronaries compared to patients with any stage of coronary disease. Conclusions: With regard to wearable devices, the EBI-derived PAT can serve as a substrate for PWV calculations and cardiovascular risk assessment, although these data require further confirmation.

## 1. Introduction

The detection, measurement, and treatment of high blood pressure (BP) as a silent killer, the leading risk factor for death and disability globally, plays an essential role in the prevention and control of cardiovascular disease (CV) [1,2,3,4,5,6,7,8]. In clinical practice, measurements of systolic and diastolic BP are performed with a conventional brachial cuff sphygmomanometer [9] to represent the pressure load in the large conduit arteries that connect to the peripheral vascular beds and organs. However, this method may have variable accuracy [10], and due to their anatomical proximity, pulsatile stress on organs such as the heart, brain, and kidneys is determined more closely by central aortic pulse pressure than by peripheral pulse pressure [11]. There is increasing evidence that central, more than peripheral, BP is associated with target organ damage and potential CV risk [12,13]. Arterial stiffness and the mechanics of arterial pulse wave propagation, reflection, and pulse pressure [14,15] are key parts of the complex, and not only the mechanical but also the neurohumoral [8,16] vascular pressure system. Various technological and computational technologies are increasingly being introduced into the field of cardiovascular disease management [17,18,19]. Hence, indirect measurement of the brachial arterial BP is the first and most simple step in describing the cardiovascular risk. The addition of the information derived from peripheral arterial pressure curves can improve the assessment of the CV function. It helps in the treatment and management of high BP and for the assessment of arterial stiffness and vascular dysfunction. Pulse transit time (PTT)-based calculation of pulse wave velocity (PWV) is one of the approaches used. Photoplethysmography (PPG) from the finger or toe can be used with simultaneous electrocardiography (ECG). Increased arterial stiffness and BP decreases PTT values and increases the PWV [3,20,21,22]. Another option is to measure peripheral arterial pulsatile volume curves with EBI to obtain continuous data with diagnostic and prognostic significance [23,24,25].

The present study aimed to achieve non-invasive monitoring of the central aortic BP using EBI sensing. Electronic circuitry with an embedded data acquisition and signal processing approach is described. The appropriate placement of electrodes is proposed, and the results of modeling concerning the best sensitivity and stability of the measurement procedures are discussed.

## 2. Materials and Methods

### 2.1. Selection and Characteristics of Patients

In this prospective single-center study, we enrolled 66 consecutive patients aged 45–75 years. All patients were referred for a scheduled coronary angiography evaluation at East Tallinn Central Hospital between 2020–2021. This study was approved by the Estonian Research Ethics Committee of the National Institute of Health Development, and the study was carried out according to the Declaration of Helsinki. Written informed consent was obtained from all participants after a complete explanation of the protocol and possible risks.

The usage of the gold standard reference method provides this study with analytical value and precision but also presents technical challenges and ethical considerations as this method is invasive; therefore, healthy patients and patients who did not have coronary angiography indications were excluded.

Individuals under 18 and over 75 years of age were excluded to reduce potential bias from age-related vascular changes. All enrolled patients were hemodynamically stable and in sinus rhythm before the procedure, presented with palpable pulses in both radial arteries, and had a contralateral variance in non-invasive brachial BP measurements of less than 20 mmHg.

A second set of specific exclusion criteria contains conditions that can influence arterial pressure and volume curves, such as an ongoing acute coronary syndrome, previous myocardial infarction, previous coronary revascularization (percutaneous coronary intervention or coronary artery bypass grafting, PCI/CABG), persistent atrial fibrillation (AF), significant and/or corrected valvular disease, cardiomyopathy with left ventricular ejection fraction (LVEF) <30%, pericardial effusion, renal insufficiency with estimated glomerular filtration rate (eGFR) <30mL/min/1.73 m^2^, and other significant and/or life-shortening vascular diseases (vasculitis, aortic disease, including endovascular thoracic endovascular aortic repair (TEVAR), or chemotherapy).

The original study plan was set to enroll 100 patients. During the study randomization period, over 2000 patients were prescreened. Due to the COVID-19 lockdown, only 66 patients were eventually enrolled out of the required 100. For further analysis, 44 patients out of 66 were applicable. Post-randomization exclusions occurred due to multiple, sometimes coinciding, reasons. Some patients who enrolled in the study met the clinical exclusion criteria after post-randomization diagnostics or after reviewing hospital records. Sometimes, exclusion diagnoses are only revealed after secondary diagnostics during the same or subsequent hospitalization, so they cannot be recognized during the original enrollment. Some exclusion criteria, like arrhythmias, only appear during coronary angiography or during recordings and, for this reason, also cannot be prevented.

Exclusions due to ECG changes (bigeminus arrhythmia, atrial fibrillation, and suspicion of acute coronary syndrome), missing echocardiography study, pericardial effusion, significant aortic stenosis, and myopericardial disease were present in 21 recordings. Erroneous EBI recordings were mainly due to a detached electrode, missing signals, or ineligible recordings. Erroneous arterial pressure records (missing signal, blood pressure line not flushed correctly, and artificial recording through contrast media) were present in 14 cases. Technical errors were present mainly in the first study phase (contrast media in arterial lines, electrode(s) not connected properly, motion artefacts during recording, etc.). The sterile operating sheet that covered the patient during the procedure occasionally caused contact variations in the electrodes, and static electricity was found to cause measurement errors. The protocol was changed, and no patient or procedure-related movements were allowed during the recording. If technical errors were detected by an experienced nurse or doctor, repetitive recording was initialized.

The decision was made to analyze only the patients who strictly met the study inclusion criteria and had existing and correct recordings.

### 2.2. Patient’s Characteristics

Hypertension was defined as >140 mmHg for systolic and/or >90 mmHg for diastolic BP in repeated measurements or if the patient was permanently treated with antihypertensive drugs. Diabetes mellitus was defined as fasting blood glucose concentration >7 mmol L^−1^ or antihyperglycemic drug treatment. Dyslipidaemia was defined as LDL cholesterol >3 mmol L^−1^ or antidyslipidemic drug treatment. The body mass index (BMI) was calculated by dividing the patient’s weight at current hospitalization in kilograms by their height in meters squared. Vascular disease was defined by a previous diagnosis of lower extremity arterial disease, aortic aneurysm, or atherosclerosis, and a history of ischemic or hemorrhagic stroke.

### 2.3. Coronary Catheterization and Measurement of the Invasive Hemodynamic Data and EBI

**Figure 1:**
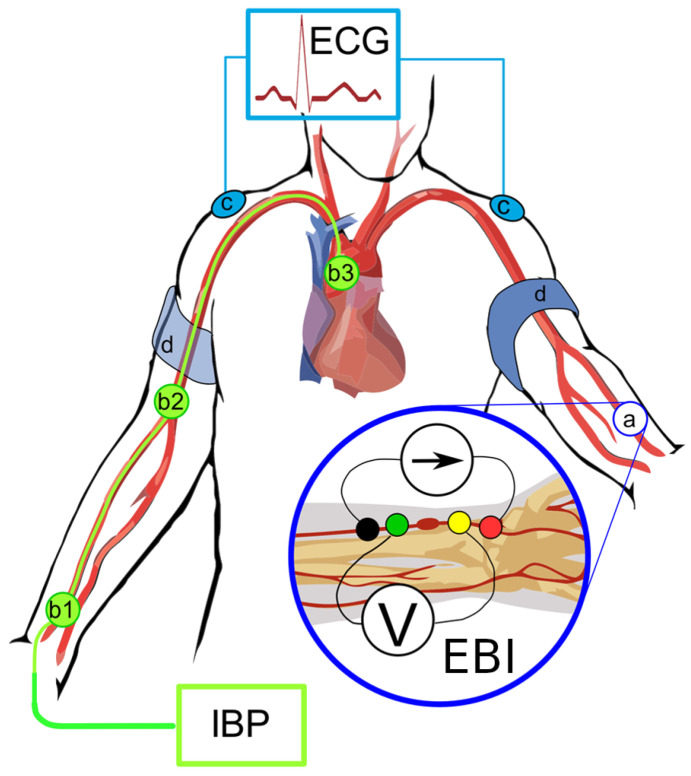
**Electrode placing and measuring points in cath lab.** IBP: invasive (arterial) blood pressure. IBP measuring points: *b1* distal radial, *b2* proximal radial/brachial (not discussed in this paper), and *b3* central supravalvular. ECG: electrocardiogram. ECG measure points: *c* points of registration. EBI: electrical bioimpedance. EBI measure points: *a* registration points and *d* position of cuff for BP measurement.

Non-invasive brachial BP measurements were performed on the cardiology ward upon arrival, and selective coronary angiography with invasive blood pressure and bioimpedance measurements were performed on the same day or the next day as scheduled. The placing of electrodes and measuring points in the catheterization laboratory are shown in Figure 1.

Hemodynamic measurements were conducted while the patient was in a supine position. For premedication, midazolam 3.5 mg p.o. was administered in the ward. In the catheterization laboratory, electrodes for electrical bioimpedance (EBI) were placed longitudinally to the left radial palpable pulse (Figure 1), and three ECG electrodes were placed on the shoulders and left leg. Sorimex EK-S 35 PSG 130 ST or equal were used for both ECG and EBI recordings. The pressure was calibrated to normal atmospheric pressure prior to inserting the catheters.

The right radial artery was punctured with the Seldinger technique. BP measurements were performed via a cannula (Terumo SURFLO IV Catheter 20 136 G, internal diameter 0.8 mm, catheter length 32 mm, flow rate 60mL/min, or equal) of the radial artery. The cannula was changed to sheath (Terumo 6F GLIDESHEATH™ 139 Hydrophilic Coated Introducer Sheath with inner diameter 0.082 in, length 25 cm, or equal) as standard procedure and the central BP was obtained via a 6F Pigtail catheter (Boston Scientific with 8 side holes, catheter internal diameter 1.4 mm, length 110 cm, or equal) inserted through the introducer sheath and positioned above the aortic annulus. Coronary angiograms were performed as standard procedure after study-related pressure measurements.

All direct invasive BP measurements were achieved via a saline (NaCl 0.9%) flushed line (ACIST CVI AngioTouch Kit), which was connected to the ACIST CVi™ Contrast Delivery System (Standard) and registered with the PHILIPS Xper PM5 hemodynamic system. Measurements of EBI were carried out simultaneously for every invasive measurement of BP (i.e., left radial EBI–right radial BP; left radial EBI—ascending aorta). All registered curves (EBI, ECG, and BP) were recorded with the MFLI Lock-in Amplifier of Zurich Instruments, Zurich, Switzerland, and saved digitally.

Coronary artery anatomy was assessed visually by obtaining physician fluoroscopic images during the standard procedure performed with the Allura Xper FD20 Clarity angiograph. Direct coronary angiography was performed via 6F diagnostic catheters with Visipaque 320 mg Iodine mL^−1^. The narrowing of the coronary arteries was categorized as follows:

0: Normal coronaries;
1: Minimal (≤25)% decrease in vessel diameter;
2: Moderate (26–50)% decrease in vessel diameter;
3: Medium (51–75)% decrease in vessel diameter;
4: Severe (76–90)% decrease in vessel diameter;
5: Preocclusion (91–99)%;
6: Occlusion (100)%.

The patients’ numeric information was stored in the hospital’s internal Red Cap database.

### 2.4. Data Analysis

The following risk factor groups were formed based on the patient’s baseline clinical characteristics: body mass index (BMI) greater than 35, diabetes mellitus, hypertension, dyslipidemia without medical treatment, current or previous smoking, and arterial vascular disease in locations other than the coronary arteries. Based on coronary lesions, four groups of patients were formed as follows (see Table 1):

1. Normal coronaries;
2. Minimal/moderate (≤50)% decrease in vessel diameter;
3. Medium (51–75)% decrease in vessel diameter, stenoses that are non-revascularization objects, and 1–2 vessel disease;
4. Significant (51–100)% decrease in vessel diameter, stenoses that are objects of revascularization, and three-vessel disease.

### 2.5. Signal Preprocessing

The acquired data were smoothed with the Savitzky–Golay filter (with a window duration of 0.1 s and polynomial order of 3) and resampled at a new sample rate equal to 150 sample s^−1^. In this case, a 0.1 s window duration was chosen to effectively capture the rapid changes in blood pressure and impedance waveforms. This duration aligns with the systolic interval of both blood pressure and impedance signals, ensuring that key features are preserved while minimizing distortions. We found that a third-order Savitzky–Golay filter provided an optimal balance between smoothing and feature preservation. It effectively reduces noise while maintaining the essential characteristics of blood pressure and EBI waveforms. The same filter parameters were applied to smooth the ECG signals. Although a 0.1 s window duration might seem large for ECG signals in general, our analysis focused solely on the R peak location in time. As a result, over-smoothed ECG waveforms did not pose a problem, and this approach guaranteed that no valuable noise distortions interfered with the extraction of the R peak timing.

**Figure 2:**
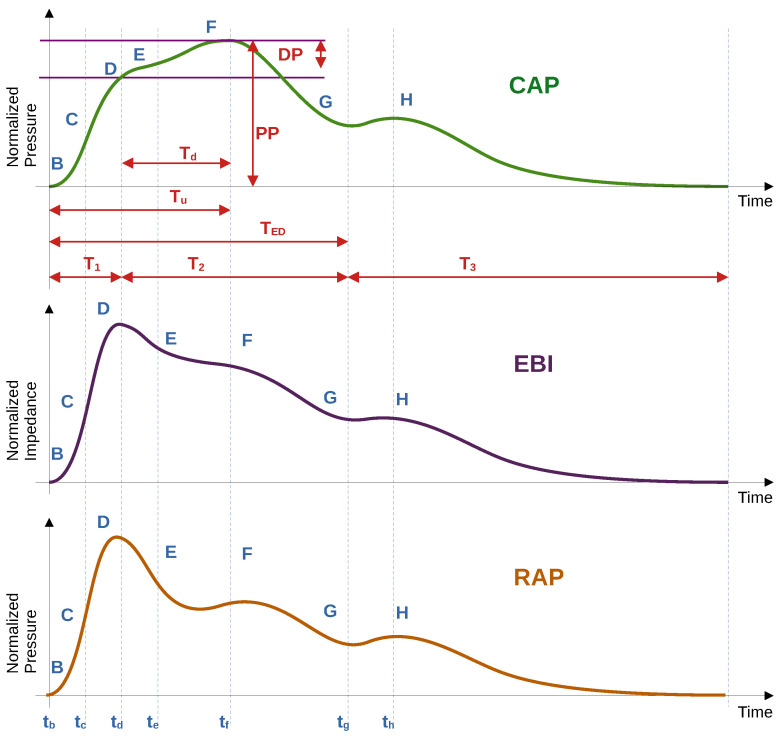
**Illustration of typical CAP, EBI, and RAP curves with fiducial points.** CAP—central arterial pressure curve: (B) start of curve, (D) inflection point, (D,E) inflection curve, (F) pressure maximum, and (G) end systolic pressure/dicrotic notch/closure of aortic valve. EBI—electrical bioimpedance curve and RAP—radial artery pressure: (B) start of curve/systole, (C) 50% time of upstroke, (D) pressure maximum, (E,F) inflection curve, (F) inflection point, (G) dicrotic notch, and (H) diastolic peak. Time intervals: $T_{1}$—inflection time, indicating the time (s) between the onset of the distally traveling (forward ejected) wave and the arrival of the proximally traveling (backward reflected) wave, $T_{u}$—systolic upstroke time, $T_{ed}$—time to end-systole, $T_{d-f}$ ($T_{d}$) inflection time, $T_{2}$—systolic duration of the reflected aortic pressure wave, and $T_{1}+T_{2}=T_{ED}$—ejection duration.

In this study, instead of subtracting the mean value (high-pass filter), baseline compensation (subtraction) was used to extract the cardiac component. The curve that passes the minima or base of the waveforms of the signals (point B in Figure 2) was taken as the baseline of both the cardiac impedance signal and the pulse wave of the blood pressure. These minima were related to the end of the diastolic and the beginning of the systolic processes. For this task, a Hankel matrix-based signal decomposer was used [26].

### 2.6. Fiducial Points Overview

In the literature, different labels for the fiducial points can be found, and these labels may vary between papers. In the current study, fiducial points were selected according to the classical description of relevant physiological events. These visually distinctive points were ordered in an alphabetical sequence of Latin letters from B to H, also corresponding to the fiducial points of the EBI/RAP and CAP waveforms. B point was used as a base point, corresponding to the baseline of the curve.

Figure 2 shows typical illustrative waveforms of the EBI and CAP periods with fiducial points, time intervals, and amplitude intervals of our interest.

Several points on the CAP and EBI/RAP curves were identified, but only five (B, C, D, F, and G) met the criteria for acceptable accuracy across all patients. Consequently, only these points were included for further analysis in this study.

The estimation of fiducial points varies between EBI/RAP waveforms and the CAP waveform. Detailed procedures for each are provided in the subsections that follow.

#### 2.6.1. EBI/RAP Fiducial Point Estimation

The following text describes the algorithms used to identify points on the EBI and RAP signal waveforms. Although the text refers specifically to impedance signals, the same principles apply to RAP signals and their corresponding points.

- **B point**:
  In cardiac impedance waveform analysis, the B point marks the beginning of the peripheral impedance curve initiated by cardiac systolic ejection. The B point detection algorithm uses a straightforward approach by identifying the first point of the signal. This initial point represents the onset of the arterial systolic curve before the rapid upstroke phase of the impedance curve. Unlike more complex feature detection methods, B point identification is based on temporal positioning rather than derivative analysis or peak detection.
- **C point**:
  The C point detection algorithm identifies the maximum slope in the cardiac impedance waveform, representing the rapid ventricular ejection phase. This critical point is mathematically determined by finding the maximum value of the first derivative dZ/dt using equation ${dZ(t)/dt|}_{\max}=\max\left(dZ(t)/dt\right)$. The C point occurs as a result of ventricular systole, usually appearing between the B point (cycle onset) and the D point (peak impedance) and serves as a clinically significant marker for the assessment of contractility.
- **D point**:
  The D point detection algorithm identifies the peak amplitude in the cardiac EBI waveform, representing the maximum blood flow during ventricular ejection. This critical cardiac feature is computationally determined by finding the global maximum value within the signal.
- **F point**:
  The F point detection algorithm identifies a critical inflection point in the descending phase of the cardiac impedance waveform, marking the beginning of reduced ejection velocity after peak systolic function. This point is computationally determined by analyzing the third derivative $d^{3}Z/dt^{3}$ of the impedance signal, specifically at the first transition from positive to negative slope after point D, as represented by the equation $F=\{t|d^{3}Z/dt^{3}<0,t>t_{D}\}$. Point F serves as a significant marker for the onset of the cardiac relaxation phase, located between the peak ejection (point D) and the beginning of diastole.
- **G point**:
  The G point detection algorithm identifies the onset of the dicrotic notch in the cardiac impedance waveform, marking the beginning of isovolumic relaxation. This inflection point is determined by analyzing the third derivative $d^{3}Z/dt^{3}$ of the impedance signal, specifically finding the most significant negative excursion after the F point according to $G=\arg\min_{t}\left(d^{3}Z/dt^{3}\right)$, where $t\in (t_{F},t_{F}\;\cdot \;k)$ and k=2.5 is a limiting factor. The G point serves as a critical marker for the onset of the ventricular relaxation phase, positioned during the early descending limb of the impedance curve after peak ejection.

#### 2.6.2. CAP Fiducial Point Estimation

Now, the techniques applied to detect the corresponding points on the CAP signal waveforms are described.

- **B point**:
  This estimates the location of point B in the CAP waveform. Point B represents the onset of the systolic upstroke in the CAP signal, corresponding to the opening of the aortic valve and the beginning of ventricular ejection. This implementation simply identifies the first point in the signal as point B, which serves as a reference point for subsequent cardiac cycle analysis.
- **C point**:
  This estimates the C point in the CAP waveform, which represents the maximum rate of pressure increase during systole. The C point is identified as the maximum of the first derivative ${dCAP(t)/dt|}_{max}$ of the pressure signal, corresponding to the steepest ascending slope of the pressure curve. Physiologically, this point reflects the rapid ejection phase and provides information about ventricular–arterial coupling. The time of occurrence and the amplitude of the C point are important markers for assessing left ventricular contractility and arterial compliance.
- **D point**:
  This estimates the D point in the central aortic pressure (CAP) waveform, which represents the completion of the deceleration of the ventricular ejection. The method analyzes the third derivative of pressure $d^{3}CAP(t)/dt^{3}$ to identify the first positive segment after point C, locating D in the first quarter of this segment. Mathematically, the location of the D point (time instance) is defined as follows: $t_{D}=t|max\left(d^{3}CAP(t)/dt^{3}\right)$ in the interval $\left[t_{C},0.95\;\cdot \;t_{F}\right]$, where $t_{C}$ is the time at point C, and $t_{F}$ is the time at point F. This approach captures the maximum rate of change in pressure acceleration during late ventricular ejection, with the search window limited between points C and F to ensure physiological relevance.
- **F point**:
  This estimates the F point in the CAP waveform. The F point represents the maximum systolic pressure in the CAP signal, occurring during the ventricular ejection phase. This method identifies F by locating the maximum amplitude value in the CAP signal F=max(CAP(t)).
- **G point**:
  This estimates the G point in the CAP waveform, which represents the onset of left ventricular ejection. The algorithm identifies G by locating the minimum of the first derivative and then finding the beginning of the first negative segment in the third derivative within a defined interval. This interval spans from the minimum of the first derivative to halfway between this minimum and the end of the signal, providing a focused search region for detecting inflection changes. This approach exploits the inflection characteristics in which the third derivative $d^{3}CAP(t)/dt^{3}$ becomes negative, indicating the acceleration change at the onset of the systolic ejection phase in the CAP signal.

### 2.7. Period Ensemble Processing

This section introduces the methodology for processing cardiac signal periods within a synchronized ensemble framework.

Cardiovascular time interval measurements are preferably carried out during the same cardiac cycle. Only one invasive catheter was used for pressure acquisition at sequential locations. ECG recording was used as a “reference key“ to align central and peripheral arterial pressure recordings in time. Peripheral EBI was recorded with both, and the best of the two recordings was selected. Also, ECG was recorded for arrhythmia verification. Cardiac rhythm disturbances invalidate pressure wave analysis for vascular stiffness diagnostics (pulse wave reflections are interrupted and thereby do not carry true information about the elasticity of distal vasculature).

The number of heartbeats per minute, variability in systolic and diastolic pressures, and volume changes are modified in various physiological situations. Emotional or physical stress triggers the release of adrenaline, which increases the heart rate and contractility, leading to higher systolic pressure. In the described study, the respiration cycle and emotional stress are the main causes of heart rate changes. Simple random sequential recordings do not provide a precise enough comparison of pressure, volume, and EBI.

Additionally, real-life conditions introduce distortions, resulting in ‘good’ signal intervals that may be relatively short and interrupted by distorted segments. Given these factors, we collected all synchronous periods and conducted an analysis of the time intervals between fiducial points. Thus, by selecting and aligning periods from various signals, such as EBI, CAP, and ECG, it was ensured that only valid and simultaneous cardiac periods were analyzed.

The measured signals were affected by noise or electrode movements, which introduced distortions. To ensure reliable analysis, only periods without corruption were selected, meaning that all segments within the ensemble (ECG, CAP, EBI, and RAP) were free from disturbances. As a result, the number of suitable periods varied between patients, depending on individual signal quality and external interference.

Subsequent steps, including outlier detection and normalization, refined the data further to create representative and averaged period ensembles. Detailed descriptions of each processing step are provided in the following subsections.

#### 2.7.1. Period Ensemble Synchronization

After all periods had been found, these periods were synchronized by storing only simultaneous periods found in all the signals (EBI, RAP, and CAP). All cardiac periods that were only present in one signal were ignored and excluded from further analysis.

The computational analysis of recorded hemodynamic curves is based on the automatic recognition of relevant points and time intervals of clinical significance on the pressure or volume curves. Curves were formed as combined ensembles of 5–15 consecutive pulses. Obtained ensembles were used for the statistical analysis.

#### 2.7.2. EBI and CAP Period Selection

After extracting the cardiac component, the period detection algorithm was applied to the impedance and BP cardiac signals.

#### 2.7.3. ECG Period Selection

**Figure 3:**
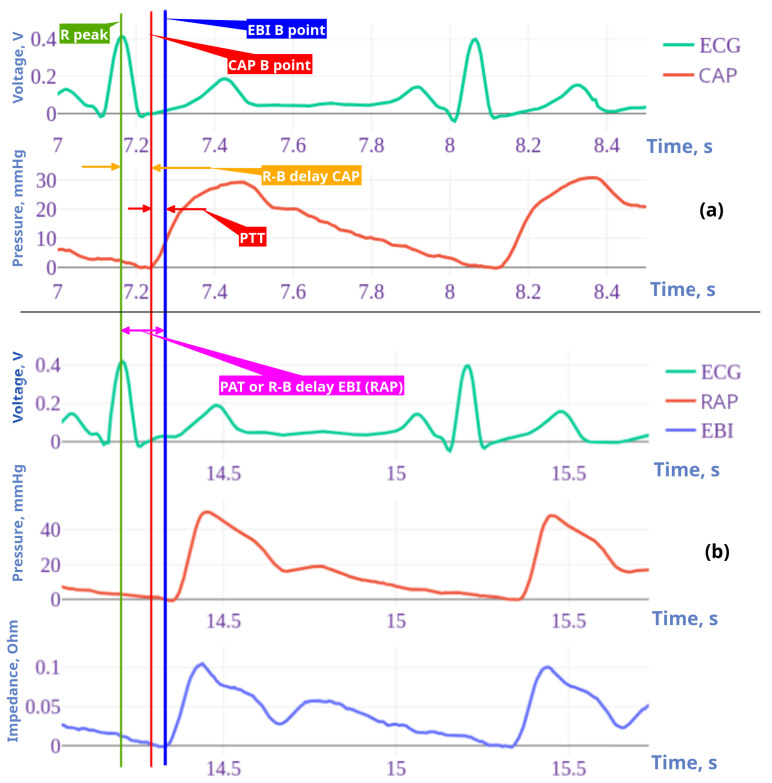
**Examples of wave recordings and time points for patient nr 3.** ECG and central pressure recording (**a**) followed by radial artery pressure and EBI recordings taken simultaneously with ECG (**b**). Timelines are shown with vertical lines as follows: green: ECG R peak, blue: radial arterial pressure (RAP) and EBI B point, and red: central arterial pressure B point. The R-B delay radial (RAP/EBI) interval and pulse arrival time (PAT) are represented by purple arrows and central (CAP) R-B delay is represented by an orange arrow. Pulse arrival time (PAT) is the time from the R peak to the peripheral (RAP B point), and the pulse transit time (PTT) is the time between the central and distal pressure wave B points (red arrow). *Note*: 2 cycle recordings are shown here; for the calculations, 5–15 cycles were used.

ECG signal periods were selected using the time frame of the impedance corresponding to the periods shifted back in time by 300 ms (to obtain the whole ECG period). The starting points of the periods (the B point in Figure 2 and Figure 3) were obtained from the first-order derivatives of the corresponding signals (EBI, CAP, and RAP). The location of the B point was defined as the time instance when the value of the signal’s first derivative $S^{\prime }$ increased above the threshold level $L_{B}=0.15\;\max\left(S^{\prime }\right)$, with $\max(S^{\prime })$ as the maximum value of the signal’s first-order derivative.

#### 2.7.4. Detection of Period Outliers

Thereafter, all saved periods were filtered from outliers by the period signal waveform. Consequently, all the periods of the selected signals (EBI, CAP, RAP, and ECG) were placed into corresponding ensembles. For each ensemble, a three-step procedure for outlier detection was introduced:

1. Filtering of periods by their lengths, with very long and very short periods excluded;
2. A PCA-based outlier detector from the pyOD Python library was used [27];
3. A COPOD-based outlier detector [28] from the pyOD Python library was used [27].

All three steps were applied sequentially. When a period was present in only one collection after outlier detection, it was excluded from further analysis.

As a result, we obtain ensembles with “suitable periods”, which were signal periods free from corruption caused by noise, electrode movements, or other measurement-related distortions. Any segments affected by such disturbances were classified as non-suitable periods or outliers, rendering them technically unsuitable for analysis. This filtering process was purely technical and was not related to the patient’s physiological state or condition.

As a result, approximately 5–15 cardiac cycles remained in each ensemble for further analysis.

#### 2.7.5. Period Normalization

After the collection of accepted periods, they were all normalized in time by resampling (for each ensemble individually) to the same duration. The duration of the normalized period was estimated as the median duration of all periods in the set. Then, the ensemble-averaged periods were obtained from the normalized period ensembles.

### 2.8. Feature Selection

In this section, the key features considered for the simplified non-invasive heart-to-arm pulse-wave velocity (PWV) analysis are outlined. Each feature was carefully selected to improve the accuracy and reliability of the proposed methodology. The features include fiducial points, time intervals, pulse arrival time (PAT), pulse transit time (PTT), and the newly proposed complementary pulse wave velocity (CPWV). These features form the foundation of the analytical framework discussed in subsequent subsections.

#### 2.8.1. Fiducial Point Features

The selected fiducial points (described in Section 2.6.1 and shown in Figure 2) B, C, F, G, and D were estimated for EBI and AP signals, and R and Q were estimated for ECG, respectively. For further analysis, time instances and values for the B, C, F, G, and D points were scaled back to the original time scale after ensemble averaging and stored in table form. The time instances of the detected points were related to the beginning of the period, presented as point B.

#### 2.8.2. Time Intervals as Features

Furthermore, delays from the R peak to the B points of the EBI/RAP and CAP signals (Figure 3) were estimated and used in the following analysis. For each simultaneous period, these delays were individually estimated. For the final analysis, the median delay was used for all ensemble periods.

#### 2.8.3. PAT and PTT as Features

The invasive pressure-based and non-invasive volume-based pulse arrival time (PAT) was calculated. For the invasive pulse arrival time (${\mathrm{PAT}}_{\mathrm{RAP}}$), the distal radial artery time was measured in seconds from the R peak in the ECG to the beginning point of the radial pressure ($RAP_{B}$), and for the non-invasive pulse arrival time (${\mathrm{PAT}}_{\mathrm{EBI}}$), the beginning point of radial bioimpedance ($EBI_{B}$) was measured, shown as point B in Figure 2 (also see Figure 3). For the pulse transit time (PTT), the ECG R peak to point B on the CAP was measured, thereby calculating the time between points $CAP_{B}$ and $RAP_{B}$ (the $EBI_{R-B}$ delay minus the $CAP_{R-B}$ delay in Figure 3).

#### 2.8.4. PWV as a Feature

The distance concerning the pigtail catheter was measured for direct PWV calculations from the aortic valve to the distal radial artery. The pressure curves above the aortic valve (CAP) and in the distal radial artery were recorded as described in the Methods section. The pulse transit time (PTT) was calculated (1) by subtracting $PAT_{CAP}$ from $PAT_{RAP}$:

(1) $$PWV=\frac{L_{av\to ra}}{PAT_{RAP}-PAT_{CAP}},$$

where PWV is the velocity of the pulse wave, and $L_{av\to ra}$ is the distance between the aortic valve and the distal radial artery, in meters. $PAT_{RAP}$ is pulse arrival time for the radial artery pressure, and $PAT_{CAP}$ is the pulse arrival time for the central aortic pressure.

#### 2.8.5. CPWV as a Feature

For the non-invasive PWV calculation, the fixed distance value, 0.8 m, was the median radial aortic length of our investigated patients and the value of $PAT_{EBI}$ in seconds was used. For this simplified non-invasive heart-to-arm PWV, a new term, complimentary pulse wave velocity (CPWV), was proposed:

(2) $$CPWV=\frac{0.8}{PAT_{EBI}}\left[m/s\right].$$

For direct pulse wave velocity calculations, both time instances for the B point of the wave should be registered at the beginning of wave propagation and at the end of the wave propagation process (e.g., close to the heart and in the radial artery). However, using the non-invasive EBI measurement in the radial artery only allows us to register wave propagation at the endpoint of the vasculature of interest. Consequently, for the proposed approach, it is assumed that the time interval from the ECG R peak to the EBI B point does not change significantly as the measurements are performed on relatively healthy subjects without cardiac arrhythmia or high pulse-rate situations such as exercise, fewer, or cardiac emergencies.

**Figure 4:**
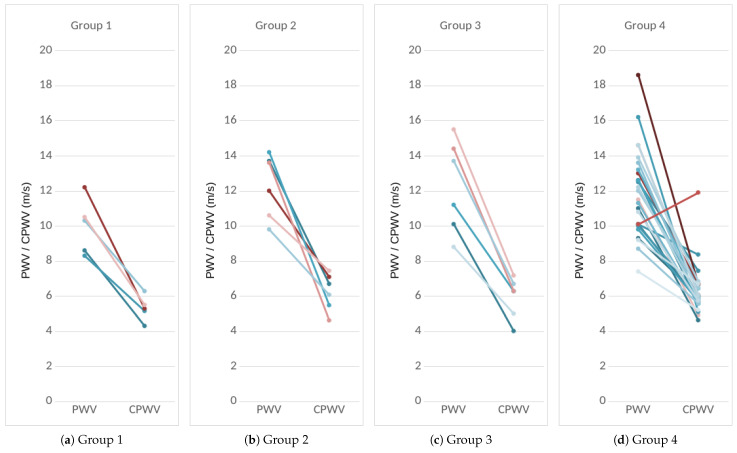
**PWV and CPWV (EBI-derived PWV) in different patient groups according to coronary disease.** Every line represents one patient. Left dots: PWV calculated from invasive radial arterial pressure RAP. Right dots: CPWV calculated from the EBI curve. (**a**) Group 1: normal coronaries, (**b**) Group 2: with coronary stenoses ≤50%, (**c**) Group 3: with coronary stenoses (51–75)%, non-revascularization objects, and 1–2 vessel disease, and (**d**) Group 4: with coronary stenoses (51–100)% that are objects of revascularization and three-vessel disease.

Therefore, we propose to use the $PAT_{EBI}$ time interval and the median arm length of the researched patients as a basis for the proposed estimate of CPWV (2). The results for CPWV together with PWV are shown in Figure 4.

## 3. Results

**Figure 5:**
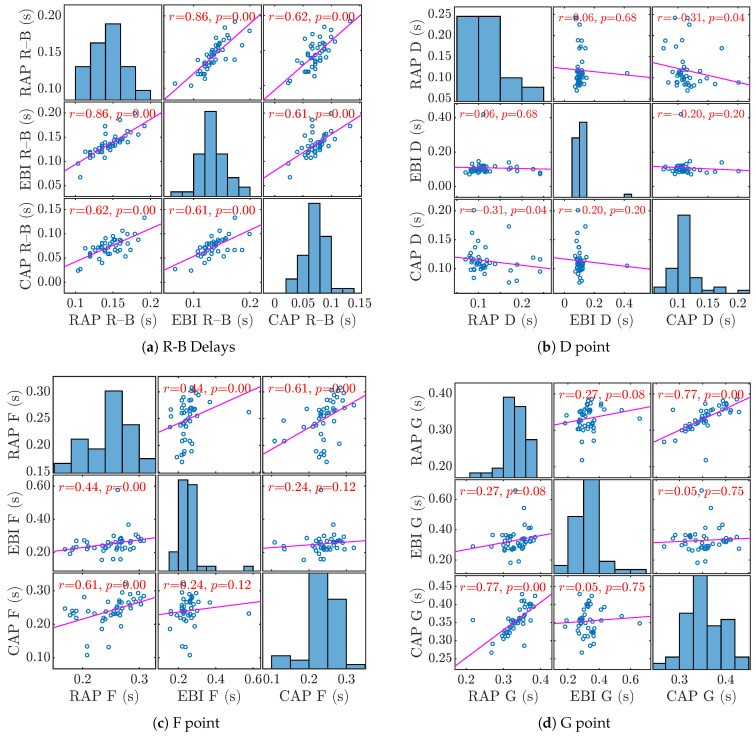
**Correlation between time points on radial and central pressures and bioimpedance curves.** B: start point of the pressure/EBI period curve, D: aortic inflection point, F: aortic pressure maximum, and G: end-systole. R is the electrocardiogram R peak. (**a**) Histograms and scatterplots of R-B delays with Spearman rank correlation coefficients and *p*-values for correlation. Spearman rank correlation coefficients for R-B delays: 0.856 for RAP-EBI R–B delay, 0.617 for CAP-RAP R-B delay, and 0.612 for CAP–EBI R-B delay; (0.7−0.9) is a high positive correlation (0.5−0.7) is a moderate positive correlation. (**b**) Histograms and scatterplots of D time points with Spearman rank correlation coefficients and *p*-values for correlation. There is no correlation or very weak correlation. (**c**) Histograms and scatterplots of F time points with Spearman rank correlation coefficients and *p*-values for correlation. There is a moderate positive correlation between RAP and CAP F-points (0.605). There is a weak positive correlation between RAP and EBI F-points (0.435) and no correlation between EBI and CAP F-points. (**d**) Histograms and scatterplots of G time points with Spearman rank correlation coefficients and *p*-values for correlation. There is a strong positive correlation between RAP and CAP G-points (0.767). There is no correlation between RAP and EBI G-points (0.635) and no correlation between EBI and CAP G-points. Corresponding Spearman correlation values for D, F, and G points are shown in Table 2. A high positive correlation is shown between RAP and CAP G points times.

In this study, data from 44 subjects were analyzed. The majority of patients were male 72.7%, the mean age of patients was 61 years (45–74 years), most of the patients were diagnosed with arterial hypertension 93.2%, 61.4% were current or former smokers, 18.2% had diabetes, and approximately 18% had hypercholesterolemia without treatment. Being overweight (BMI over 35) and additional arterial disease were both present in 9.1% patients. The baseline demographic and clinical characteristics of the study patients are presented in Table 1. Pulse wave velocities between the aortic valve and the distal radial artery were calculated, as described in the section on methods. For the statistical analysis in the paper, the Spearman rank correlation coefficient was used due to the small number of patients. The correlations between the D, F, and G time points on the pressure and bioimpedance curves are presented in Figure 5 and Table 2, where a strong correlation can be found between the times of the RAP and CAP G points.

A strong positive correlation (0.844) was found between the R-B delay of the RAP and EBI curves. R-B delay is the time from the R point of ECG and B point of RAP and EBI curves (in Figure 5a, study patients are presented as numbers).

**Figure 6:**
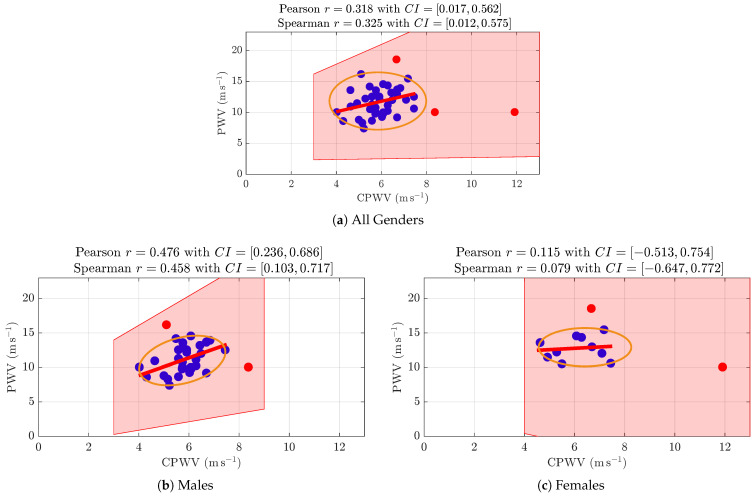
**PWV and CPWV correlations (a) for all genders, (b) for males, and (c) for females.** (**a**) A Spearman correlation coefficient 0.325 with a confidence interval of (0.012, 0.575) indicates a weak positive correlation (with outliers (red points) removed). (**b**) The Spearman correlation coefficient for the male group is 0.458 with a confidence interval of (0.103, 0.717), indicating a moderate positive correlation (with outliers removed). (**c**) The female gender group was too small to make suggestions.

**Figure 7:**
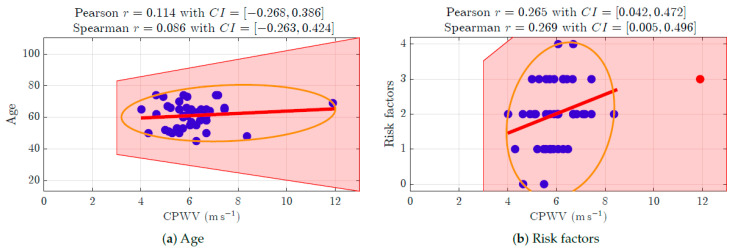
**Spearman correlation coefficient for (a) patients’ age and (b) for the number of risk factors.** (**a**) The Spearman correlation coefficient for age and CPWV is 0.086 with a confidence interval of (−0.263, 0.424). (**b**) For a number of risk factors (0–4 coexistent risks for this patient group), the CPWV correlation coefficient is 0.269 and the confidence interval is (0.005, 0.496), which indicates a weak positive correlation.

PWV calculations based on invasive (radial artery pressure, RAP) and non-invasive (radial electrical bioimpedance, EBI) R-B delay (Figure 6) showed a moderate positive correlation with the Spearman correlation coefficient 0.3255 and confidence interval (0.0160, 0.5904), and a similar correlation is shown in the male gender group (Figure 6b). The female group (Figure 6c) was too small to make suggestions. In the studied group of patients (aged 45–74 years with moderate to high cardiovascular risk), PWV was not affected by age (Figure 7a).

We pre-described six groups of common cardiovascular risk factors such as hypertension, smoking, diabetes mellitus, overweight with BMI > 35, and atherosclerosis in other vascular territories (stroke, peripheral arterial disease, etc.). Hypercholesterolemia was considered a risk factor if not treated because of the vasoprotective effect of statins when used in hyperlipidemia treatment. In the studied group, only two patients had none of these risk factors, only two had four risk factors, and there were no patients with five or six risk factors (Table 1). The correlation between risk factors and CPWV in the studied patient groups, shown in Figure 7b, was weak, with a Spearman correlation coefficient of 0.2688 and a confidence interval of (0.0121, 0.4926).

**Figure 8:**
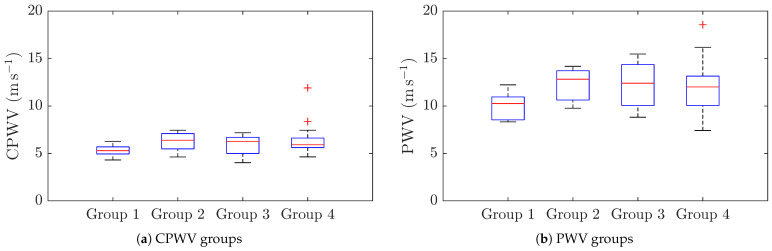
**CPWV (a) and PWV (b) ranges and medians in patient groups with different stages of coronary disease.** Patients with coronary lesions present with higher CPWV (**a**) and PWV (**b**) values compared to patients with normal coronaries. There is a moderate difference between the median values of group 1 and groups 2–4, where the *p*-value is 0.0404 for CPWM. CPWV: complimentary pulse wave velocity and PWV: pulse wave velocity. Patient groups: Group 1: normal coronaries; Group 2: with coronary stenoses ≤50%; Group 3: with coronary stenoses (51–75)%, non-revascularization objects, and 1–2 vessel disease; Group 4: with coronary stenoses (51–100)% that are objects of revascularization and three-vessel disease. The red plus sign denotes outliers, the blue box denotes 1st and 3rd quartiles, and the red line denotes the median.

Based on coronary lesions, we formed four groups of patients: the first group with normal coronaries, the second group with minimal/moderate changes (≤50% stenoses), the third group with moderately significant coronary lesions (51–75% stenoses), non-revascularization objects, and 1–2 vessel disease), and the fourth group with significant (51–100% stenoses) that are objects of revascularization and three-vessel disease (Figure 4 and Figure 8a).

Patients with mild to significant coronary lesions, as shown in Figure 4, present with higher PWV values compared to patients with normal coronaries (median values 12 m s^−1^ and 10 m s^−1^ accordingly, and p= 0.0404). The same pattern but with lower velocities can be observed with CPWV (median values for patients with coronary disease (6–6.4) m s^−1^ and 5.3 m s^−1^ for patients with normal coronaries. The difference of CPWV means it is statistically significant with a *p*-value of 0.0448 and Bayes factor of BF10: 288. Therefore, there is very weak evidence that means of group one with normal coronaries and other groups with any level of coronary disease are different (Figure 8).

Therefore, we propose to use the $PAT_{EBI}$ time interval and the median arm length of the researched patients as a basis for the proposed CPWV estimate (2). The results for CPWV and PWV are shown in Figure 4 and Figure 6.

## 4. Discussion

Blood pressure values and their pressure curve characteristics are not constant through the arterial tree, they become modified as they travel away from the heart towards the periphery, whereas the changes between central and peripheral pulse pressure not only depend on the propagation characteristics of arteries [3,15] but also on the microvasculature [29,30] and cardiac rate [31]. These influences generate circulus vitiosus, also called the ‘amplifier hypothesis’ [32]. Understanding physiological mechanisms and the difference between central and peripheral systolic pressure and pulse contour is significant in relating the conventional cuff measurements on the arm to the CV function. To determine the vascular parameters that complement the conventional BP measurement by the cuff sphygmomanometer, various methods of pulse detection have been developed, which utilize waveform features for the estimation of arterial elastic properties [3]. The non-invasive assessment of the central BP has become widely available and officially recognized in recent years [12]. Although the available techniques have limitations, it has been accepted that they may provide further complementary data to the peripheral BP regarding the management of arterial hypertension and CV risk [14]. In the transmission of blood, arterial BP, ECG, and pulse wave signals reflect the different functions of the cardiovascular system and blood flow information [33]. According to the arterial wave propagation theory, the start and end point signals of the arterial path are extracted, and PTT can be calculated and used to determine CV functional status, such as BP, arterial stiffness, and arterial compliance [33]. The present study focused on a correlation analysis, which was conducted to estimate the arterial wave propagation theory using the EBI method and explore its effectiveness in monitoring vascular tone. Technical aspects of bioimpedance curve calculations have previously been described in our work [26], and PWV is the gold standard for measuring vascular stiffness and estimating cardiovascular risk [21]. Traditionally, assessments are made on the carotid–femoral axis, which is costly, inconvenient for routine ambulatory measurements, and may cause complications if performed invasively. Additionally, this measurement becomes fused in the presence of lower-extremity arterial disease [34]. For these reasons, in this study, the aortic valve to arm axis was chosen to exclude most of the aorta and lower-limb arteries, and invasive pulse wave measurements with non-invasive ones delivered from EBI recordings were compared. Invasive methods are still considered the gold standard; so, for reference, a common cardiac catheterization pathway that begins with the right distal radial artery and follows the vasculature back to the aortic valve was used. The EBI method provides a more comfortable, cuffless measurement and is more suitable for continuous monitoring. Patient comfort and activation are important factors and have an impact on the overall recovery process [35] and safety [36]. Nevertheless, the potential changes in the EBI curve with mild to moderate physical exercise and potentially volume-changing conditions such as hypo- and hypervolemia, infection, etc., must be investigated closely before adapting the technique to cardiovascular screening methods.

EBI-derived measurements offer a non-invasive approach to assessing cardiovascular parameters, making them safer and more convenient than traditional invasive techniques. However, they come with certain limitations, particularly in the context of clinical variability and patient movements. One key limitation is sensitivity to motion artifacts. EBI measurements rely on impedance changes, which can be significantly affected by patient movements, electrode displacement, or variations in skin–electrode contact. These disruptions can introduce noise, reducing the accuracy and reliability of the measurements, especially in dynamic clinical settings. Another challenge is the dependency on tissue properties. Unlike invasive methods that directly measure blood pressure or volume, EBI estimates physiological parameters based on electrical impedance variations. Factors such as tissue conductivity, hydration levels, and electrode placement can influence readings, leading to potential variability between patients. Despite these limitations, EBI remains a valuable tool, particularly for continuous monitoring and when minimizing patient risk is a priority. The updated figures emphasize that while EBI and RAP signals may exhibit similar waveform characteristics, they originate from different measurement principles (impedance versus pressure), highlighting the inherent differences in data interpretation.

The true length of the pressure recording catheter can be used for the calculation of the invasively acquired PWV. The catheter is inserted from the distal measurement point and placed just above the aortic valve. EBI measurements on the contralateral wrist (corresponding to radial artery puncture regions) and ECG recordings from the chest were performed for the non-invasive acquisition of the PWV. The mean invasively established distance was used in the calculations. The path to the right wrist is slightly longer because it includes an extra segment: the brachiocephalic trunk before branching to the subclavian artery. In practice, the difference is usually of the order of a few centimeters and is often clinically negligible. We succeeded in demonstrating strong correlations between RAP and EBI R-B delays (that can also be defined as PAT), based on the fact that the invasive RAP curve start point (B) could be replaced by a non-invasive EBI start point and used in non-invasive PWV calculations. Although D, F, and G points seemed to be well defined in the pressure and impedance curves, there was no correlation for point D in any of the curves. Points F and G had better correlations on the central and distal invasive pressure curves, but distal invasive and non-invasive curves had only weak positive correlations for point F. This could be due to erroneous placement on the recorded curves or true differences between peripherally invasive and non-invasive pulse recordings. Thereby, D, F, and G point analysis requires further investigation; after the verification of the correct placement on all central and peripheral curves, the series of comparative RAP and EBI recordings on healthy individuals and with different potentially arterial stiffness and intravascular volume-affecting situations should be obtained. The tested method faces multiple technical difficulties. EBI signals can be severely distorted due to movement, and filtering the distance measured during the stressful invasive procedure may not be the most precise. Furthermore, single-channel recorded ECGs can be problematic for computational analysis, and even healthy subjects may not have laterally uniform pulse wave propagation. Some of these problems are discussed in the text below.

We used the Savitzky–Golay filter to smooth signals during filtering and the Hankel matrix-based signal decomposer to extract the cardiac component from the raw signals.

Distance measurement techniques and their accuracy for PWV calculation are at great variability [37]. We chose the aortic–arm axis as the carotid–femoral axis can be more affected by the subject’s height [38] and vessel elongation. For direct measuring, we considered a possible error of ±2 cm, which is acceptable if an otherwise standardized method for measurement is used, including catheter distal end placement verification by fluoroscopy. The anatomical differences between arteries of the arms could result in different BP values [39] but not PAT [40]. The pitfall of measuring BP or PAT in one arm is missed arterial occlusion or significant stenosis and, thereby, erroneous reading. This can easily be overcome by first measuring the BP in both arms, as suggested by the guidelines [41] and conducted in our study. Not only does every 5 mmHg difference identify higher cardiovascular risk [42], but over 30 mmHg may indicate subclavian arterial occlusion [43]. If, in this situation, pulsatile radial flow is present and measured, it would be collateral flow, including subclavian steal syndrome through intracranial circulation [44] and measuring PWV may be misleading. Thereby, patients with significant inter-arm pressure should be managed differently and are not subjects of screening methods such as PWV measurement.

For distal arterial waveform analysis, we recorded EBI on the left wrist as an investigation method and for the right radial invasive pressure curve for reference, both with ECG recordings. Different arterial and pressure curve characteristic points were identified according to previous authors [3,45,46,47] and named in alphabetical order from B to H to differentiate our own computational analysis points from classical points, which is a similar practice to other authors [23,45]. In this study stage, we only used these fiducial points to describe the pressure curves and verify the EBI curves. Further analysis will be the subject of subsequent studies.

The computational analysis of the recorded hemodynamic curves was based on automatic recognition of certain points and time intervals of clinical significance of pressure or volume curves. We were able to show correlations between the D, F, and G time points on the pressure and bioimpedance curves and a strong correlation between the RAP and CAP G points. We also showed a strong positive correlation between the R-B delay of RAP and EBI curves, confirming that the radial invasive pressure curve could be replaced by a bioimpedance-based curve measurement to generate non-invasive measurements.

There is considerable heterogeneity required between measurement sites and techniques to obtain the waveform for PWV calculations [48]. To define PAT, we used the most definable and also the most used ECG R peak [49] instead of the R onset and volume wave (EBI) curve beginning/onset (EBI-B) as PAT (onset) is preferred over PAT (peak) due to its higher physiological correlation [49]. PAT is not only a good surrogate marker for PTT but contains more information, such as the time delay between the electrical depolarization of the left ventricle of the heart and the opening of the aortic valve, known as the pre-ejection period (PEP) [50]. For these reasons, we used PTT for invasive PWV calculations and PAT for EBI-derived PWV calculations and proposed the term CPWV for PAT-derived non-invasive PWV to distinguish between the two terms.

PWV increases normally with age; its mean values are less than 6 m s^−1^ in the patients’ twenties and more than 8 m s^−1^ in subjects over 60 years old [51]. For increased cardiovascular risk, common threshold values for arterial stiffness are >10 m s^−1^ for carotid–femoral PWV and >14 m s^−1^ for brachial–ankle PWV [2]. The presence of an isolated PWV > 13 m s^−1^ is a strong predictor of cardiovascular mortality [52]. As PTT is shorter than PAT, the values of CPWV are considerably shorter than PWV values; therefore, new cutoff values for different risk groups are required.

We could only show a moderate difference between CPWV in different groups of patients. One reason may be the absence of a truly healthy group and a relatively homogeneous cardiovascular risk group since all patients were considered high-risk and scheduled for coronarography. It is also known that vascular atherosclerosis begins with soft plaque formation and positive remodeling long before visual changes appear using coronarography [53]; furthermore, not all stenoses cause cardiac events, a situation called visual–functional mismatch [54]. For the same reason, our third and fourth groups start with 51% lesions and are differentiated by operators’ opinions on whether they are objects of revascularization or not. Therefore, more sophisticated methods for intravascular diagnostics, for example, intravascular ultrasound (IVUS) with lipid core detection by near-infrared spectroscopy (NIRS) [55] and FFR with IMR [56], could be used to describe vascular damage and regroup patients. By grouping individuals according to the true stages of atherosclerotic, CPWV, and other characteristics of bioimpedance, the arterial curve can be more precisely calibrated, possibly offering significant value in preventive medicine.

## 5. Conclusions

Good quality and artifact-free recordings are required for the analysis of the EBI waveform, the measurement of the PAT, and the calculation of the PWV. It is aided by the detection of changes during follow-up. Complementary quantifiable information on the arterial pulse waveform can provide a markedly improved means of non-invasive characterization of CV function and better stratification of CV risk [3] and could lead to innovative clinical applications developed in collaboration with the medical device industry and used in various cardiovascular fields, including ambulatory and patient self-management settings, as well as in hospital and acute care environments.

Monitoring peripheral EBI variations is a promising method that has the potential to replace invasive or burdensome techniques for cardiovascular measurements. With regard to wearable devices, EBI-derived PAT can serve as a substrate for blood pressure calculations or cardiovascular risk assessment, although these data require further confirmation.

However, the present study has some limitations. First, the study was conducted on a relatively small number of patients. Second, the control group with normal coronaries cannot be defined as a reference or healthy group because visual estimation of coronaries can significantly over- and underestimate real vascular pathology. Third, several aspects of data acquisition and signal processing require further refinement. A thorough investigation is required for the optimal placement of the electrodes, more efficient suppression of different measurement artifacts, and the optimal selection of measurement time points and sequence lengths, to name a few.

## Figures and Tables

**Table 1:** Demographic and clinical data of the patients. Total number of studied patients is 44 with a mean age of 61 years and a median age of 63 years. Risk factors: body mass index (BMI) greater than 35, diabetes mellitus, hypertension, dyslipidemia without medical treatment, current or previous smoking, an d vascular disease in locations other than the coronary arteries. *Notes*: (^1^): not revascularization objects, (^2^): revascularization objects, and CAD: coronary artery disease.

	Number	%
**Gender:**		
male	32	72.7
female	12	27.3
**Age:**		
45–54	11	25.0
55–64	15	34.1
65–74	18	40.9
**Risk factors:**		
hypertension	41	93.2
dyslipidemia with NO treatment	8	18.2
BMI >35	4	9.1
current/former smoking	27	61.4
diabetes	8	18.2
vascular pathology	4	9.1
**Risks levels:**		
**0** risk factors	2	4.5
**1** risk factor	10	22.7
**2** risk factors	19	43.2
**3** risk factors	11	25.0
**4** risk factors	2	4.5
**5** risk factors	0	0.0
**6** risk factors	0	0.0
**CAD stages:**		
**1**: normal coronaries	5	11.4
**2**: minimal/moderate (≤50%) decrease in vessel diameter	6	13.6
**3**: medium (51–75)% decrease in vessel diameter ^1^	6	13.6
**4**: significant (51–100)% decrease in vessel diameter ^2^	27	61.4

**Table 2:** Spearman correlations between selected time points concerning radial and central pressures and bioimpedance curves.

	RAP D Time	EBI D Time
**RAP D time**		
**EBI D time**	0.078	
**CAP D time**	−0.3128	−0.2191
	**RAP F time**	**EBI F time**
**RAP F time**		
**EBI F time**	0.3002	
**CAP F time**	0.6249	0.0701
	**RAP G time**	**EBI G time**
**RAP G time**		
**EBI G time**	0.10223	
**CAP G time**	0.8587	0.0981

## Data Availability

The datasets generated and/or analyzed during the current study are not publicly available due to protection of personal data.

## Abbreviations

The following abbreviations are used in this manuscript:

AF	Atrial Fibrillation
AP	Arterial Pressure
BMI	Body Mass Index
BP	Blood Pressure (non-invasive)
CABG	Coronary Artery Bypass Grafting
CAD	Coronary Artery Disease
CAP	Central Aortic Pressure (invasive)
CFR	Coronary Flow Reserve
COPOD	Copula-Based Outlier Detection
CPWV	Complimentary Pulse Wave Velocity
CV	Cardiovascular
EBI	Electrical Bio-Impedance
ECG	Electrocardiogram
ED	Ejection Duration
FFR	Fractional Flow Reserve
IBP	Invasive Blood Pressure
IMR	Index of Microvascular Resistance
IVUS	Intravascular Ultrasound
LDL	Low-Density Lipoprotein (cholesterol)
LVEF	Left Ventricular Ejection Fraction
MFLI	Multi Frequency Lock-In
NIRS	Near-Infrared Spectroscopy
NaCl	chemical formula of Sodium chloride
PAT	Pulse Arrival Time
PCA	Principal Component Analysis
PCI	Percutaneous Coronary Intervention
PEP	Pre-Ejection Period
PPG	Photoplethysmography
PTT	Pulse Transit Time
PWV	Pulse Wave Velocity
RAP	Radial Artery Pressure (invasive)
TEVAR	Thoracic Endovascular Aortic Repair
eGFR	Estimated Glomerular Filtration Rate
pyOD	Python Outlier Detection (Python language library)
